# *Brevis plant1*, a putative inositol polyphosphate 5-phosphatase, is required for internode elongation in maize

**DOI:** 10.1093/jxb/erv554

**Published:** 2016-01-14

**Authors:** Luis M. Avila, Diego Cerrudo, Clarence Swanton, Lewis Lukens

**Affiliations:** Department of Plant Agriculture, University of Guelph, 50 Stone Road East, Guelph, Ontario N1G 2W1, Canada

**Keywords:** Brachytic, *br3*, *brevis plant1*, *bv1*, cell elongation, development, growth, internode elongation, plant height.

## Abstract

In maize (*Zea mays* L.), as in other grass species, stem elongation occurs during growth and most noticeably upon the transition to flowering. Genes that reduce stem elongation have been important to reduce stem breakage, or lodging. Stem elongation has been mediated by dwarf and brachytic/brevis plant mutants that affect giberellic acid and auxin pathways, respectively. Maize *brevis plant1* (*bv1*) mutants, first identified over 80 years ago, strongly resemble *brachytic2* mutants that have shortened internodes, short internode cells, and are deficient in auxin transport. Here, we characterized two novel *bv1* maize mutants. We found that an inositol polyphosphate 5-phosphatase orthologue of the rice gene *dwarf50* was the molecular basis for the *bv1* phenotype, implicating auxin-mediated inositol polyphosphate and/or phosphoinositide signalling in stem elongation. We suggest that auxin-mediated internode elongation involves processes that also contribute to stem gravitropism. Genes misregulated in *bv1* mutants included genes important for cell wall synthesis, transmembrane transport, and cytoskeletal function. Mutant and wild-type plants were indistinguishable early in development, responded similarly to changes in light quality, had unaltered flowering times, and had normal flower development. These attributes suggest that breeding could utilize *bv1* alleles to increase crop grain yields.

## Highlight

We report two novel reduced-height maize mutants caused by mutations within a putative inositol polyphosphate 5-phosphatase-encoding gene. Auxin-mediated internode elongation probably requires regulation of inositol and/or phospholipid metabolism.

## Introduction

In grasses including *Zea mays*, plant height at maturity largely results from stem elongation (Moore *et al.*, 1991). This elongation occurs primarily after the shoot apical meristem initiates the production of reproductive tissues in response to developmental and environmental signals (Siemer *et al.*, 1969; Colasanti and Muzynski, 2009). Prior to the reproductive transition, the shoot apex is below or near the surface of the soil. New leaves emerge from the pseudostem, a whorl composed of the leaf sheaths of younger leaves. For example, when eight leaf tips have emerged from the maize whorl, the shoot meristem can be below ground. Cell division and cell expansion within the internodes extend the shoot apex through the whorl. At maturity, the apex terminates with the mature tassel.

Tall crop plants can have poor agronomic performance due to lodging, especially in soils with a high nitrogen content. Several reduced-height mutants have been utilized in cereal breeding. For example, the semi-dwarfing gene *sd1* was used in the rice semi-dwarf cultivar IR8, which produced record yields in Asia (Monna *et al.*, 2002; Spielmeyer *et al.*, 2002). Similarly *reduced height1* (*rht1*) in wheat and *dwarf-3* in sorghum have increased plant yields by reducing stem lodging (Peng *et al.*, 1999; Khush, 2001; Barrero Farfan *et al.*, 2012). In maize, one class of reduced-height mutants including *anther ear1* (*an1*), *dwarf plant1* (*d1*), *dwarf plant2* (*d2*) *dwarf plant3* (*d3*), *dwarf plant5* (*d5*), *dwarf plant8* (*D8*), and *dwarf plant11* (*D11*) are deficient in gibberellic acid (GA) biosynthesis or signalling. A second class, including *brachytic1* (*br1*), *brachytic2* (*br2*), and *brevis plant1* [*bv1*, formerly termed *brachytic3* (*br3*)] are gibberellin insensitive. GA mutants have stunted growth habits very early in development and the mutations affect organ size and sex determination (Phinney, 1956; Fujioka *et al*., 1988a, b; Bensen *et al.*, 1995; Spray *et al.*, 1996; Wang *et al.*, 2013). Flowers may be bisexual, thereby affecting seed production. In contrast, the phenotypes of the brachytic/brevis plant mutants are less pleiotropic than those of the anther ear/dwarf mutants. These mutants, known for over half a century, can have leaves similar in size to wild-type leaves and inflorescences with normal, unisexual male and female flowers (Li, 1931; Kempton, 1941; Multani *et al.*, 2003; Pilu *et al.*, 2007), and are thus candidates to reduce the plant height of cultivated maize.

Molecular characterization of reduced-height maize mutants has identified genes involved in GA or auxin biosynthesis and signalling. For example, *DWARF1* and *DWARF3* genes encode a gibberellin 3-oxidase and a cytochrome P450, respectively, enzymes important for GA biosynthesis, (Winkler and Helentjaris, 1995; Chen *et al.*, 2014). *Brachytic2* (*Br2*) encodes an ATP-binding cassette phosphoglycoprotein (Multani *et al.*, 2003). BR2 facilitates indole-3-acetic acid (IAA) transport across the nodal/intercalary meristem region of the maize stem (Multani *et al.*, 2003; Knöller *et al.*, 2010), analogous to the function of ABCB1 within *Arabidopsis thaliana* (Noh *et al.*, 2001; Geisler *et al.*, 2005).

Here, we have described two novel *bv1/br3* mutants. At flowering, mutant plants are one-quarter the height of wild-type plants, and the internodes have shortened, rectangular pith cells. Nonetheless, the plants are indistinguishable from wild-type plants prior to the floral transition. *bv1* mutants have the same flowering time and leaf numbers at maturity as wild-type plants and respond in a similar way to changes in red:far-red (R:FR) light ratios. We found that *bv1* encodes an inositol polyphosphate 5-phosphatase (5PTase) with WD40 domains. The two mutant genotypes had non-synonymous substitutions within conserved residues of the inositol phosphatase domain, indicating that inositol polyphosphate and/or phosphoinositide signalling is necessary for auxin-mediated cell elongation. Auxin and phosphoinositide/inositol polyphosphate signalling are known components of the stem gravitropic response. Auxin-responsive gene upregulation within the mutant plants suggested the presence but misdistribution of auxin. In addition, genes involved in the production of cell wall and cytoskeleton proteins were misexpressed in the mutant, suggesting perturbation of vesicular transport. Although we did not find evidence of use of *bv1* in the past use in plant breeding, we suggest that allelic variation of the gene may contribute to future plant improvement.

## Materials and methods

### Plant materials

Two maize plants with reduced height and short internodes, similar to *br1*, *br2*, and *bv1* maize mutants, were identified within two M2 or M3 segregating families originating from the seed of a B73 maternal parent crossed with a B73 paternal parent whose pollen underwent ethyl methanesulphonate (EMS) mutagenesis as described by Till *et al.* (2004). Seed was provided by Dr Nathan Springer (University of Minnesota, MN, USA). The two mutants were termed 1301 and 1302. Both the maize B73 inbred and a wild-type sibling of 1302, whose progeny did not segregate for the reduced-height phenotype (termed 1302-Sib), were used as wild-type controls.

### Red:far-red ratio treatments

Maize plants were grown in the summer of 2011 at the Arkell research station in Guelph, Ontario, Canada, to investigate the effects of neighbouring plants on mutant and wild-type plant heights. Plants were grown in a hydroponic system using 20 l buckets filled with Turface and watered three times daily with a nutrient solution composed of 800g of 28-14-14 NPK fertilizer, 800g of 15-15-30 NPK fertilizer, 400g of NH_4_NO_3_, 60g of Micronutrient Mix (Plant Products Co., Canada) and 800g of MgSO_4_.7H_2_O dissolved in 20 l of water, and administered in a 100:1 water:solution mix. B73 wild-type and 1302 plants were grown in Turface only (high R:FR) or in Turface covered with grass sod (low R:FR) from germination until anthesis and silking (Rajcan *et al.*, 2004). The sod changed the R:FR ratio to which growing plants were exposed. The ratio was 0.76±0.02 for Turface alone and 0.30±0.02 with sod (Page *et al.*, 2010). The water, nutrient supply, and growing medium of the grass sod were separate from the maize plants. The experiment was a factorial design with two factors: genotype (levels: 1302 and B73) and treatment (levels: sod and no sod) with four replicates for each genotype×treatment combination. Each replicate of the four genotype×treatment combinations had nine buckets, each bucket with two plants. Plant height and number of leaf tips were recorded at 11 time points from 11 to 76 d after planting (leaf tips 3 to 21). The data were analysed using the R aov function, which fits linear models with categorical variables (R Core Team, 2014).

### Mapping and allelism tests of reduced-height mutants

Seed of homozygous *br1*, *br2*, and *bv1*/*br3* (referred to here as *bv1-1*) maize mutants was obtained from The Maize Genetics Cooperation Stock Center located at the University of Illinois Urbana/Champaign (stock ids: 113C, 117A, and 506L respectively). We crossed the 1301 and 1302 mutants to inbred lines A619 and Mo17 to generate F_2_ mapping populations. Mapping populations were grown in Guelph, Ontario, Canada. In the summer of 2009, a small mapping population consisting of 73 F_2_ plants of a cross between 1302 and A619 was grown. In the summers of 2010 and 2011, 2122 and 1325 plants of the F_2_ of a cross between 1301 and the inbred Mo17 were grown in the same location. Finally, in 2012, tissue from 491 individuals of the F_2_ of 1301×Mo17 with the *bv1* phenotype were collected. For each population, the heights of individual plants were scored at maturity and classified as tall or short.

Genomic DNA was extracted from leaf tissue samples of F_2_ plants, using either a GeneElute^TM^ Plant Genomic DNA Mini prep kit (Sigma, St Louis, MO, USA) or a CTAB-based protocol adapted in-house for 96-well plate extractions. An initial genotyping of 73 individuals was done at the Analytical Genetics Technology Centre of the Princess Margaret Hospital in Toronto, Canada, using the iPLEX Gold Assay (Sequenom, San Diego, CA, USA) using 68 polymorphic single-nucleotide polymorphisms (SNPs) between B73 and A619 over the 10 maize chromosomes (Gore *et al.*, 2009). For fine mapping, the F_2_ plants grown in years 2010, 2011, and 2012 were genotyped with simple sequence repeat (SSR) markers and SNP markers. SSR markers used included umc1060, umc1966, umc1747, umc1155, umc1822, and bnlg609 from MaizeGDB (http://www.maizegdb.org/ssr.php). PCR for genotyping with SSR markers was performed under the following conditions: 1min of initial denaturation at 95 °C, 35 cycles of 30s denaturation at 95 °C, 30s annealing at 55 °C, and 40s extension at 72°C, followed by 10min of final extension at 72 °C. Additional SNPs (Gore *et al.*, 2009) were assayed using custom allele discrimination TaqMan probes designed with the software PrimerExpress 3.0 (Applied Biosystems, Foster City, CA, USA) following the manufacturer’s protocol.

### RNA extraction, pooling, sequencing, and analysis

Plants of genotypes 1301, 1302, 1302-Sib, and B73 were grown following a completely randomized design with four plants per genotype, in a greenhouse in 20 l buckets, using Sunshine Growing Mix #4/LA4 (Sun Gro Horticulture Canada Ltd) as medium and the same nutrients solution as described above. At 14 leaf tips (eight mature leaves with blades fully emerged from whorl with a visible base of an exposed leaf blade, V8), 40 d after planting, approximately 5mm^3^ samples of stalk tissue from the sixth internode of each plant (counting from the lowest visible internode) were stored in 1.5ml of RNAlater (Qiagen GmbH, Hilden, Germany). At this stage of development for inbred B73, the shoot apical meristem has transitioned into the production of male reproductive structures. Elongation initiates sequentially, with lower internodes initiating growth prior to the upper internodes (Robertson 1994). Internode six elongation has just commenced at the V8 stage. At the time of sampling, the wild-type and mutant plant sixth internodes were each about 1mm in length. Internodes beneath internode 6 were visibly longer in the wild-type plants than in the mutant plants. Samples were kept overnight at 4 °C and then stored at –20 °C. Total RNA of individual samples was isolated using TRIzol (Invitrogen, USA) and purified using a Qiagen RNeasy Mini kit column according to the manufacturer’s instructions. RNA (3 μg) from the four plant samples of each genotype were pooled. cDNA libraries were constructed and sequenced at the Clinical Genomics Center of the Mount Sinai Hospital in Toronto, Ontario, Canada, following the Illumina TruSeq Sample Preparation Guide (Part # 15008136 Rev. A) and using an Illumina Hiseq 2500 system. Paired-end reads of 100bp were aligned with the B73 reference genome (ZmB73 RefGen_v2) using TopHat v.2.0.9 (Schnable *et al.*, 2009; Trapnell *et al.*, 2012). Aligned reads were assembled into putative transcripts with cufflinks (v.2.1.1), and cuffdiff (v.2.1.1) identified significant transcript abundance differences between genotypes (Trapnell *et al.*, 2012). Differences in transcript abundance were considered significant when the Benjamini–Hochberg corrected *P* values were lower than the default false discovery rate (FDR) of 0.05 (Benjamini and Hochberg, 1995; Trapnell *et al.*, 2012). The number of paired-end reads obtained for 1301, 1302, 1302-Sib, and B73 was 23 993 453, 20 109 205, 23 993 453, and 15 566 118, respectively, with 75.6, 77.9, 75.8, and 81.0% concordant pair alignment rates, respectively. Transcript abundances were quantified and normalized to fragments per kilobase of exon per million fragments mapped (FPKM ).

Translated coding sequence similarity searches were performed against the UniProt database (Jain *et al.*, 2009), and alignments were visualized in the software Geneious Basic v.5.6.4 (Meintjes *et al.*, 2012). SNPs relative to the B73 reference genome were identified from aligned RNA sequencing (RNA-Seq) reads using samtools mpileup and filtered with bcftools (Li *et al.*, 2009) for occurrences when the estimated allele frequency of the putative SNP was equal to 1 (AF1=1) and read depth was >8 (DP>8).

The Integrative Genomics Viewer (Robinson *et al.*, 2011) was used to visualize RNA-Seq alignments and manually confirm SNPs. Gene orthologues were identified with information from Gramene (http://www.gramene.org/, release #38, accessed August 2013) and UniProt (http://uniprot.org/, accessed August 2013).

### Light microscopy

Transverse and longitudinal sections of fresh tissue were taken from the middle of internodes 1, 2, and 8 of 35-, 63-, and 91-d-old 1302 and B73 plants. Fresh tissue samples were sectioned with a razor blade and stained using 0.05% toluidine blue solution. The height and width of individual cells in longitudinal sections were estimated using ImageJ software.

## Results

### Genetic and phenotypic characterization of novel *bv1* alleles

**Fig. 1.**
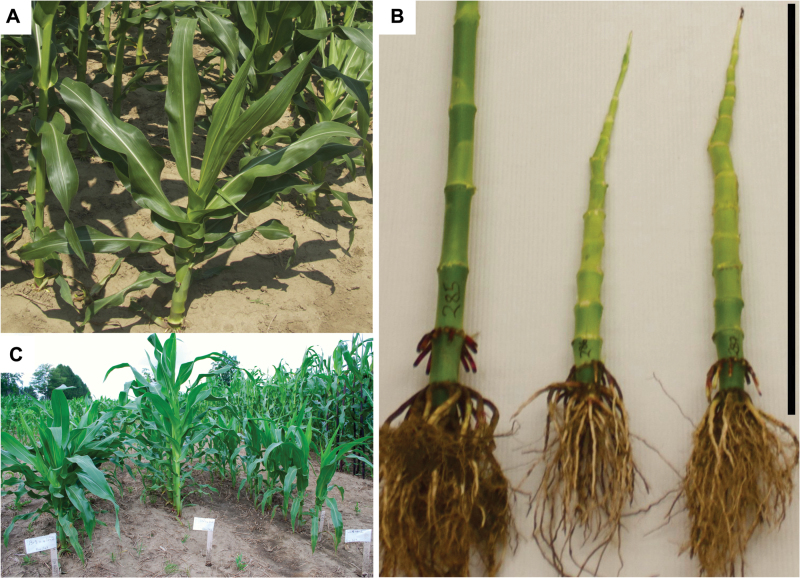
Maize mutants 1301 and 1302 have reduced height and short internodes compared with wild type plants. (A) An F_2_ *bv1*/*bv1* plant in the field 63 d after planting from a *bv1-1301* mutant×Mo17 cross. (B) Internodes of B73 (left), *bv1-1302* mutant (middle), and *bv1-1301* mutant (right), 66 d after planting. Leaves were removed. Bar 50cm. (C) F_1_ progeny of *bv1-1* mutant×1302 (left), *br2* mutant×1302 (middle), and 1301×1302 (right) 55 d after planting.

**Fig. 2.**
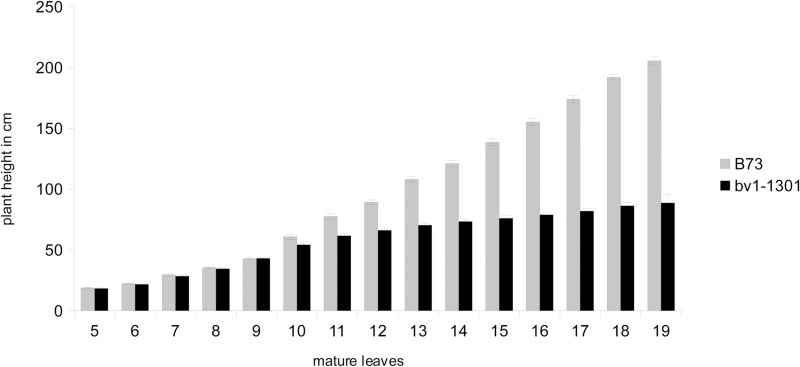
Plant heights of greenhouse-grown 1301 and B73 plants measured when leaf ligules from five to 19 leaves had emerged (V stage). Error bars represent the standard error (*n*=16).

We identified two reduced-height maize mutants termed 1301 and 1302 in two families segregating for EMS-induced mutations. Self-pollination of both mutants generated populations composed entirely of reduced-height plants. At anthesis in the field, 1301 and 1302 mutants were 48 and 50cm, compared with 191cm for wild-type plants (Figs 1A and 2). The sizes of 1301 and 1302 mutant plant leaves were similar to wild type, and tassels and ears developed normally, although the ears were notably smaller. The ratio of wild-type:reduced-height plants (57:16) within an F_2_ population (from self-pollinated 1302×A619 hybrids) was consistent with the 3:1 segregation ratio expected for a monogenic recessive allele (χ^2^ test, *P*>0.05). Hybrids between 1301 and 1302 mutants failed to complement, suggesting the same gene in both genotypes caused the mutant phenotype. The progeny of crosses between both mutants (1301 and 1302) and *br2* mutant was wild type. The progeny of 1302 and *bv1-1* (formerly *br3*) had reduced height (Fig. 1C). We concluded that the reduced-height genotypes were each homozygous for different novel *bv1* alleles: *bv1-1301* and *bv1-1302*. The wild-type sibling plant of the *bv1-1302* mutant is referred hereafter as *Bv1-1302.*

Plant heights of *bv1-1301* and *bv1-1302* mutants were similar to wild-type heights early in development. In both greenhouse and outdoor bucket experiments, heights of *bv1-1301*, *bv1-1302*, and B73 plants were almost identical when a few mature leaves were visible (Fig. 2). In B73, the primary meristem transitioned into an inflorescence meristem when eight mature leaves had emerged from the whorl. The difference in plant height between mutant and wild-type plants was significant when 10 leaves had emerged from the whorl (V10) (Fig. 2).

Low ratios of red:far-red light and auxin treatment can induce cell expansion (Wolbang *et al.*, 2004), and we investigated whether these factors affected plant height differently within the *bv1* mutants compared with wild-type controls. A low R:FR treatment induced similar increases in plant height in *bv1-1302* mutants as in wild-type B73 plants. Throughout early development, the plant heights of both mutant and wild-type plants grown in the low R:FR environment were 1–2cm greater than plants not exposed to low R:FR (see Supplementary Fig. S1 at *JXB* online). In addition, in both wild-type and mutant genotypes, leaf emergence occurred more quickly among the plants not exposed to the low R:FR treatment than in those exposed. Beginning 30 d after planting, at approximately the V4–V5 stage, low R:FR-treated plants had up to one less emerged leaf tip than plants not exposed to low R:FR conditions (see Supplementary Fig. S2 at *JXB* online). Thus, the low R:FR treatment-induced increase of plant height and decrease of leaf emergence rate were not *bv1* dependent. Consistent with the similar response of wild-type and mutant plants to changes in the R:FR ratio, spraying *bv1-1301* mutant plants and wild-type plants with IAA did not alter the plant heights of either genotype (data not shown).

**Fig. 3.**
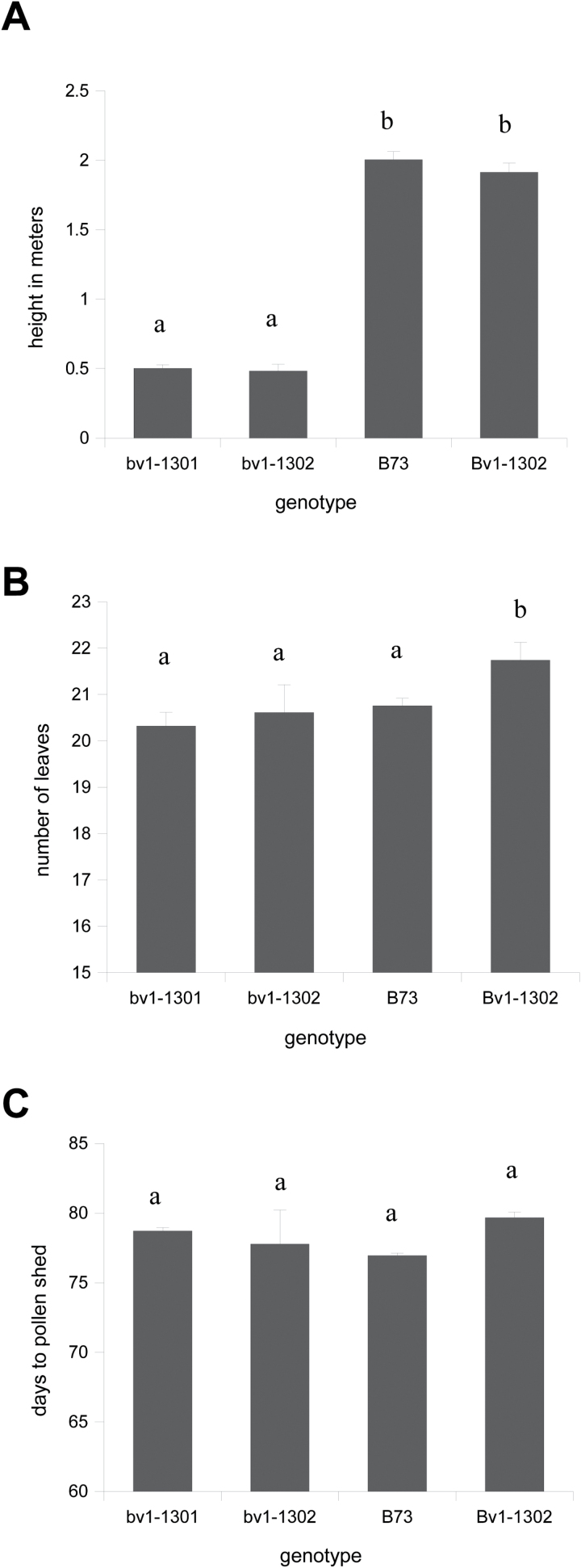
Mean plant height (A), number of leaves (B), and days to pollen shed since planting (C) of field-grown 1301, 1302, Bv1-1302, and the B73 inbred, measured at anthesis. Three rows of 16 plants were grown per genotype in a completely randomized design. Bars with different letters were significantly different (*P*=0.05; *n*=3). Error bars represent the standard error.

**Fig. 4.**
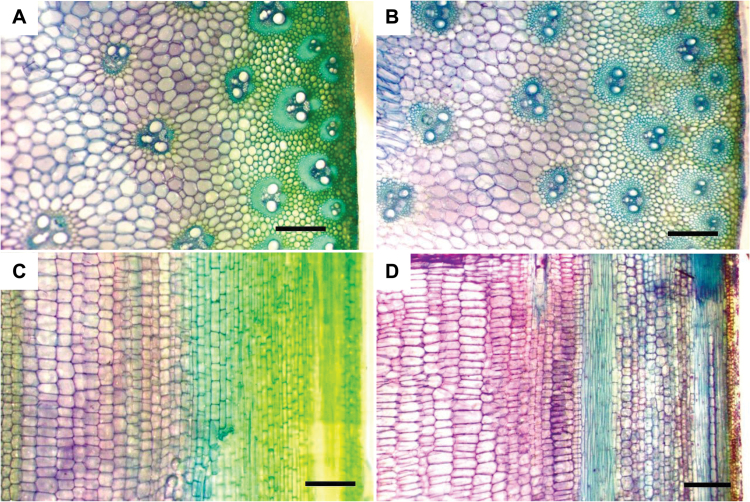
Transverse and longitudinal stalk sections of B73 (A, C) and *bv1-1302* mutant (B, D) internode 8. Cells of *bv1-1302* parenchyma cells had an average height of 66.2 vs 89.5 μm for B73 cells (standard error 3.9 and 3.4 μm, respectively, *n*=30). Both parenchyma and vascular cells are visible in the four panels. Cells of *bv1-1302* were wider than B73 cells (178.9 vs 125.9 μm, respectively; standard error 5.6 and 4.9, respectively, *n*=30). Samples were taken 91 d after planting after the tassel had fully emerged from the whorl. Bars, 400 μm.

Although both upper and lower internodes of *bv1-1301* and *bv1-1302* mutants were short relative to wild-type plants, the biggest effect of *bv1* was on the upper internodes (Fig. 1A, B and Supplementary Fig. S3 at *JXB* online). At V14, when the tassel had emerged from the whorl, the maximum internode lengths of *bv1-1301* and *bv1-1302* mutants were 5.08 and 4.13cm, respectively (Supplementary Fig. S3). In contrast, the maximum B73 internode length was 14.23cm. Lengths of internodes 1 and 2 did not significantly differ (Supplementary Fig. S3). From internodes 3 to 8, the differences between wild-type internodes and mutant internodes increased. Lengths of internodes 8 –14 differed the most between mutant and wild-type plants. The differences varied from 9.2 to 11.9cm for *bv1-1301* mutants and from 9.9 to 12.0cm for *bv1-1302* mutants (Supplementary Fig. S3). These short internodes resulted in a severely reduced plant height at maturity (Fig. 3A). Among plants harvested after the tassel had fully emerged from the whorl, internode 8 parenchyma cell architectures in transverse sections were similar between mutant and B73 plants (Fig. 4A, B). We also noted no differences in cell morphology when comparing transverse and longitudinal sections from internodes 1 and 2 (data not shown). However, in longitudinal sections of internode 8, mutant plant parenchyma cells were short and rectangular relative wild-type cells (Fig. 4C, D).

Flowering time is often highly positively correlated with plant height and leaf number (e.g. Khanal *et al.*, 2011). However, at maturity, *bv1-1301*, *bv1-1302*, and B73 all had similar leaf numbers and flowering times (*P*>0.05) (Fig. 3B)*. Bv1-1302* developed one more leaf (*P*<0.05) than the other genotypes with no significant difference in days to anthesis (Fig. 3C)). The number of leaves between B73 and mutant plants did not significantly differ at any sampled developmental stage (Supplementary Fig. S2). These results showed that stem elongation can be independent of plant developmental timing.

### *bv1-1301*, *bv1-1302*, and *bv1* have mutations within the same locus, which maps to a gene encoding a putative 5PTase

**Fig. 5.**
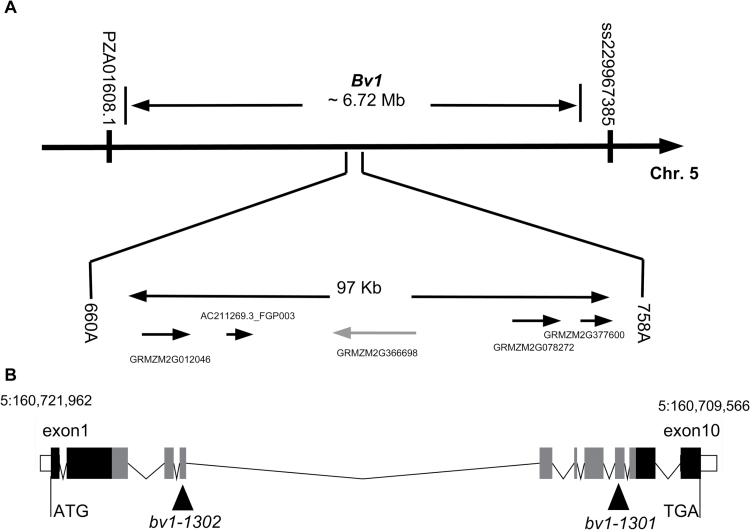
(A) The *bv1* locus genetically maps between SNP markers PZA01608.1 and ss229967385 with physical positions 158 599 491 and 165 319 744, respectively, on chromosome 5. Fine mapping of *bv1* to an ~97kb region using SNP markers 660A and 758A with physical positions 160 660 813 and 160 758 241 (based on markers ss229964467 and ss229964577 from the HapMap project; Gore *et al.*, 2009). The arrows represent the genes and their transcription orientation for this region, according to the B73 genome (AGPv2 release 5b.60). In grey is the candidate gene GRMZM2G366698. (B) Exons (solid boxes) and introns (lines) of transcript GRMZM2G366698_T01. The inositol phosphatase domain is shown in grey spanning from exon 2 to exon 9 (IPPc pfam PF03372, aa 249–595). White boxes are untranslated regions. Mutation points in *bv1-1301* and *bv1-1302* alleles are shown with arrowheads.

We assayed 491 F_2_ mutants and 3447 mutant and wild-type F_2_ plants of a *bv1-1301*×Mo17 cross to map *bv1-1301* between nt 160 660 813 and 160 758 241 (~97kb) on the ZmB73 RefGen_v2 pseudochromosome 5 long arm (Fig. 5A and Supplementary Fig. S4 at *JXB* online). This map location coincides with the interval on chromosome 5 to which Beavis *et al.* (1991) genetically mapped *bv1* and is less than 2Mb away from the region in the IBM2 2008 Neighbors map estimated for *bv1* (5:158 262 770–159 134 168 between markers IDP4992 and IDP2401, available on MaizeGDB). Eight F_2_ plants had recombinant genotypes at the SNP markers flanking the ~97kb interval (Supplementary Fig. S4). There were five uncharacterized genes in this region (ZmB73_5b_FGS): GRMZM2G012046, AC211269.3_FGP003, GRMZM2G366698, GRMZM2G078272, and GRMZM2G377600. *bv1-1301* mutant and Mo17 SNPs within GRMZM2G012046 and GRMZM2G377600 defined the outer boundaries of the ~97kb interval, and we observed recombinants between a SNP position within the gene and the mutant phenotype for both genes (Fig. 5). GRMZM2G366698 is a homologue of the rice *dwarf50* gene (*d50*; Sato-Izawa *et al.*, 2012). *d50* (NCBI UniGene: Os.18140, Os02g0477700) is the closest rice homologue to GRMZM2G366698 (Protein BLAST against NCBI database, GenBank locus: BAJ61820.1, 99% query cover, e-value 0.0, 79% identity) and is located on rice chromosome 2: 16 360 196–16 364 326, a region syntenic to maize chromosome 5. Maize genes GRMZM2G012046 and GRMZM2G078272 flanking GRMZM2G366698 also had homologous genes in the same region of rice chromosome 2, Os02g0475300 (2:16, 259, 553–16, 264, 489) and Os02g0478550 (2:16, 426, 109–16, 430, 574), respectively. The putative gene spanned ~13kb and had three annotated splice variants, GRMZM2G366698_T01 (2648bp, 766 aa), GRMZM2G366698_T02 (2664bp, 213 aa), and GRMZM2G366698_T03 (3236bp, 308 aa). The putative GRMZM2G366698_P01 protein encoded a putative 5PTase. It contained an inositol polyphosphate phosphatase catalytic domain SMART: SM00128 (Letunic *et al.*, 2012) and a Trp-Asp signature WD40 repeat-containing domain that may create a platform for stable protein–protein interactions (Ananieva *et al.*, 2008; Stirnimann *et al.*, 2010). *bv1* has a similar phenotype to four *d50* rice mutants (Sato-Izawa *et al.*, 2012). We discovered two indel markers flanking GRMZM2G366698 (see Supplementary Table S1 at *JXB* online). Among the eight plants with recombinants within the ~97kb interval (Supplementary Fig. S4), seven wild-type individuals were heterozygous across the locus 1301/Mo17, and the one brachytic mutant was homozygous 1301/1301 for this region. Thus, the height phenotype and GRMZM2G366698 genotype were completely linked across more than 3900 F_2_ plants. We did not determine whether any of the other four genes within the interval were also completely linked with the *bv1* phenotype.

We performed RNA-Seq analyses of RNA harvested from the sixth internodes of wild-type and mutant plants shortly after the shoot had transitioned to generating reproductive tissues. Within the ~97kb interval, transcript abundances were similar between the three mutant and wild-type genes for which we detecteded transcripts (GRMZM2G366698, GRMZM2G012046, and GRMZM2G078272; *P*>0.05). The mean coverage of each gene’s transcript across genotypes was 15×, 0.51×, and 13×, respectively. Cufflinks predicted six different isoforms of GRMZM2G366698 within B73, Bv-1301, and the 1302 and 1301 *bv1* mutants. These were the three splice variants reported in the reference genome annotation and three additional potentially novel splice variants (see Supplementary Tables S2 and S3 at *JXB* online). Although the isoform with highest abundance differed across genotypes (Supplementary Table S2), these isoform differences are likely stochastic. Across our sample of 22 446 expressed genes (FPKM >1), the most highly expressed isoform differed in 4223 (18.8%).

**Fig. 6.**
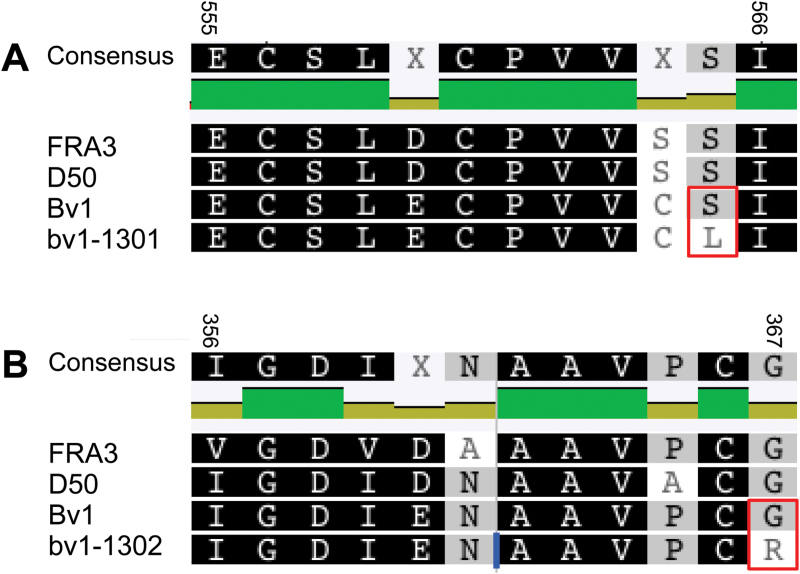
A section of the protein alignments of Arabidopsis FRA3, rice D50, maize BV1 (as in the B73 reference genome), and maize mutants *BV1-1301* (A) and *BV1-1302* (B). The amino acid changes caused by EMS-induced base transitions are marked with a rectangle. Numbers indicate the amino acid positions in GRMZM2G366698_P1.

Reads derived from *bv1-1301* mRNA reveal a G-to-A transition (on physical position 5:160 710 789) causing an S-to-L change at position 565 of 766 in GRMZM2G366698_P01 (Figs 5B and 6A). This nucleotide was found in 28 out of 28 RNA-Seq reads (10 unique reads) that overlapped this position. Transcript reads of *bv1-1302* RNA identified a C-to-T (on physical position 5:160 716 570 ZmB73 RefGen_v2) transition in exon 4 that caused a putative G-to-R amino acid change at position 367 of GRMZM2G366698_P01 supported by 34 out of 34 RNA-Seq reads for this region (24 unique reads) (Figs 5B and 6B). Transcript reads of Bv1-1302 plants had only Cs at 5:160 716 570. Amino acid positions 367 and 565 were both within the inositol phosphatase domain of GRMZM2G366698. Amino acids at position 367 were invariant across diverse monocots and dicots. The amino acid at position 565 only differed within a single *Ricinus communis* homologue (see Supplementary Fig. S5 at *JXB* online). Using Sanger sequencing of amplified genomic DNA, we obtained 99% of all intronic sequences of GRMZM2G366698 from *bv1-1301* and *bv1-1302* plants and found no nucleotide changes relative to the B73 reference genome. We confirmed the *bv1-1301* allele through Sanger sequencing, but were unable to amplify GC-rich exon 10, the location of the *bv1-1302* mutation, from any genotype. Among the three expressed genes in the ~97kb interval, we identified no other nucleotide differences between *bv1-1301*, *bv1-1302*, B73, and *Bv1-1302* reads and the B73 reference genome. We concluded that point mutations in GRMZM2G366698 caused the reduced-height phenotypes of *bv1-1301* and *bv1-1302* mutants.

Although Sato-Izawa *et al.* (2012) noted that the putative protein encoded by the *d50* gene has the highest homology with *A. thaliana FRAGILE FIBER3* (*FRA3*; AT1G65580), a gene necessary for stem mechanical strength and proper cell wall development, we noted that *bv1* and *d50* were as similar to the putative *A. thaliana* inositol polyphosphate 5 phosphatase proteins At5PTase12 (At2g43900), At5PTase13 (At1g05630), and At5PTase14 (At2g31830) as they were to *FRA3*. In protein BLAST searches, over 97% of the *bv1* and *d50* protein sequences overlapped these putative Arabidopsis proteins, all with over 51% identity. For example, 99% of GRMZM2G366698_P01 aligned with FRA3 with 53% identity, and 97% of GRMZM2G366698_P01 aligned with At5PTase13 with 54% identity. At5PTase12 (At2g43900) and At5PTase13 (At1g05630) exhibited phosphatase activity towards Ins(1,4,5)P3, while At5PTase14 (At2g31830) and FRA3 exhibited phosphatase activity towards Ins(1,4,5)P3 and PtdIns(3,4,5) but had the highest affinity for PtdIns(4,5)P2 (Zhong *et al.*, 2004; Zhong and Ye, 2004). The similarity of *bv1* to At5PTase13 was especially interesting because At5PTase13 mutants have altered auxin accumulation and distribution, altered expression of several genes responsible for auxin biosynthesis and transport, and altered vesicle trafficking that may affect auxin transport (Lin and Wang, 2005; Wang *et al.*, 2009).

Of 256 and 108 genes differentially expressed in *bv1-1301* and *bv1-1302* internode tissue relative to B73 internode tissue, respectively (FDR-adjusted *P*<0.05; see Supplementary Table S4 at *JXB* online), 42 genes were differentially expressed in the two mutants relative to B73 and were not differentially expressed between a *Bv1/Bv1 bv1-1302* sister-line and B73 (see Supplementary Table S5 at *JXB* online). We assigned putative functions to 16 of these genes (Table 1). Genes with higher transcript levels in mutant plants compared with wild-type plants included: (i) GRMZM2G180659, which encodes a putative lysine histidine transporter; (ii) GRMZM2G099467 with similarity to a gibberellin 20 oxidase 2/flavonol synthase/flavanone 3-hydroxylase encoding gene; (iii) GRMZM2G063917 and (iv) GRMZM2G118345, both putative phenylalanine ammonia-lyase (PAL)-encoding genes; (v) GRMZM2G072529, encoding a putative aminocyclopropanecarboxylate oxidase (ACC oxidase), a key enzyme for the biosynthesis of ethylene (Kieber and Ecker, 1993); (vi) GRMZM2G127789, a glutathione *S*-transferase (GST 29); and (vii) GRMZM2G04902, an isochorismatase family protein rutB. Genes with lower transcript levels in mutant than in wild-type internode tissue included: (i) GRMZM2G118786, which encodes a putative actin-related protein 2/3 complex subunit 3; and (ii) GRMZM2G103273, which encodes a β-galactosidase. Functional relationships among genes can be inferred from co-expression. We tested for enrichment of the 27 differentially expressed genes (out of 68 genes only 27 were represented in the co-expression modules study; Supplementary Table S5) within groups of genes co-expressed across maize development (Downs *et al.*, 2013). Nine (33.3%) differentially expressed genes were in one module, Zm_mod07. The nine genes included putative gibberellin 20 oxidase 2, LHT1, and PAL-encoding genes, as well as a putative endochitinase, GRMZM2G129189 (Table 1). Of the 15 527 expressed genes in Downs *et al.* (2013), 916 (5.9%) were in the Zm_mod7 module, a significantly smaller proportion (Table 1 and Supplementary Table S5, χ^2^=36.6, *P*<0.001). Because genes that are differentially expressed between wild-type and mutant plants are co-regulated across development, BV1 probably affects a discrete biological process that operates in diverse tissues. Genes within Zm_mod07 are more highly expressed in root tissues of different ages (*P*=0.0002) and mature leaf (*P*=0.003) relative to other tissues (Downs *et al.*, 2013). Among the genes most overrepresented in Zm_mod07 are those involved in amine transmembrane transporter activity, glycolipid binding, glycolipid transporter activity, and glutathione transferase activity (Downs *et al.*, 2013).

**Table 1.** *Genes differentially expressed in bv1-1301 and bv1-1302 relative to B73, with putative function annotation (values in FPKM*)

Function	Gene ID^*a*^	Putative function annotation	B73	*Bv1-1302*	*bv1-1301*	*bv1-1302*	Module membership; Score in module^*b*^
Actin polymerization	GRMZM2G118786	Actin-related protein 2/3 complex subunit 3; uncharacterized	0.90	1.31	0.00	0.00	
Cellular component	GRMZM2G180659	LHT1; lysine histidine transporter	2.27	2.83	36.45	27.36	Zm_mod07;0.82
Hormones biosynthesis	GRMZM2G099467	Gibberellin 20 oxidase 2; uncharacterized	3.28	1.81	81.31	43.81	Zm_mod07;0.70
	GRMZM2G072529	ACC oxidase	0.00	0.00	2.28	1.78	
Phenylpropanoid biosynthesis	GRMZM2G118345	Phenylalanine ammonia-lyase	2.62	3.34	21.66	16.10	Zm_mod07;0.71
	GRMZM2G063917	Phenylalanine ammonia-lyase	0.00	0.00	8.59	2.83	Zm_mod05;0.62
Metabolic process	GRMZM2G103273	β-Galactosidase 5	3.39	5.94	0.00	0.00	
	GRMZM2G049021	Isochorismatase family protein rutB	12.61	0.00	42.65	39.69	
	GRMZM2G129189	Endochitinase PR4 putative uncharacterized protein	0.00	0.00	2.82	1.93	Zm_mod07;0.59
Signalling	GRMZM2G127789	Glutathione *S*-transferase GST 29	0.00	0.00	4.16	2.81	
Transcription factor	GRMZM2G169149	WRKY62-superfamily of transcription factors having WRKY	0.00	0.00	10.87	3.91	
Transport	GRMZM2G153920	Sorbitol transporter; uncharacterized	0.00	0.00	1.15	0.98	Zm_mod04;-0.49
Transposon	GRMZM2G021020	Transposable element	22.24	22.90	0.00	0.00	
	GRMZM2G008283	Transposable element	15.17	33.16	0.0	0.0	
	GRMZM2G020508	Transposable element	4.44	4.11	12.96	13.87	
	GRMZM2G306371	Transposable element	2.53	5.26	0.0	0.0	

^*a*^ Although 42 genes were differentially expressed between *bv1-1301* and *bv1-1302* mutants relative to B73 and were not differentially expressed between Bv1-1302 and B73, 16 had functional annotations.

^*b*^ Module membership refers to the co-expression module in which the gene is classified. The score refers to the correlation of the gene's expression level with the module’s eigengene. Gene module memberships and scores are from a transcriptome analysis of diverse maize tissues (Downs *et al.*, 2013).

## Discussion

We have presented four independent lines of evidence to support GRMZM2G366698 as Bv1/Br3. First, the *bv1-1301* mutant mapped to a five-gene-containing, ~97kb chromosomal region on chromosome 5 (Fig. 5A). Fine mapping of the mutants was challenging. Gore *et al.* (2009) reported that this locus has low crossover frequency relative to other genomic regions, and we identified only eight crossovers between *bv1* and flanking markers in a survey of over 3900 F_2_ individuals. Secondly, among all F_2_ plants, the GRMZM2G366698 genotype perfectly associated with plant height. Thirdly, GRMZM2G366698 encoded a putative 5PTase and was orthologous to the rice *d50* gene, which is necessary for internode elongation. Fourthly, both mutants were homozygous for missense substitutions at highly conserved or invariant residues within the gene’s inositol phosphatase domain (Figs S5). One missense mutation converted serine to leucine in *bv1-1301*, and a second missense mutation converted glycine to arginine in *bv1-1302*. Among genes expressed within internodes and that were in the ~97kb interval, we detected no additional nucleotide differences between wild-type and mutant genes. Mutant and wild-type internodes have similar *bv1* transcript abundances.

Auxin is a signal for cell elongation, and the developing grass inflorescence exports auxin to stem nodes and internodes to promote elongation (Wolbang *et al.*, 2004). Protein(s) encoded by GRMZM2G366698 probably exhibit phosphatase activity towards the soluble inositol polyphosphate inositol 1,4,5 triphosphate [Ins(1,4,5)P3] and possibly phosphoinositides, including PtdIns(4,5)P2 (Zhong and Ye, 2004; Zhong *et al.*, 2004). We propose that BV1 plays a central role in phosphoinositide/inositol phosphate auxin signal transduction in the maize stem. As noted above, the *br2* gene encodes a P glycoprotein auxin transporter, and the mutant is defective in auxin transport (Multani *et al.*, 2003; Knöller *et al.*, 2010). The *bv1* mutant is visually similar to the *br2* mutant, and like the *bv1* mutant, *br2* mutants are insensitive to GA (Multani *et al.*, 2003). In other contexts of plant growth, 5PTases affect auxin-associated cell expansion. Stems of maize and other grasses that are placed parallel to the ground will grow upward in a gravitropic response. This bending is caused by elongation of cells at the basal side of the pulvinus, a disc-shaped region of tissue immediately apical to the stem node. Auxin levels on the top of the pulvinus are lower than at the base (Wolbang *et al.*, 2007), and auxin transport from the cells at the top of the pulvinus promote elongation of cells at the base of the pulvinus (Yun *et al.*, 2006). PtdIns(4,5)P2 is generated by phosphatidylinositol 4-phosphate 5-kinase (PIP5K). In the initial responses of maize pulvini to gravity, PIP5K activity and Ins(1,4,5)P3 levels increase in the lower pulvinus (Perera *et al.*, 1999). In *A. thaliana*, inositol signalling is also implicated in the auxin-mediated gravitropic response (Perera *et al.*, 2006; Wang *et al.*, 2009; Mei *et al.*, 2012; Ischebeck *et al.*, 2013).

The *bv1* mutant internodes may have different phosphoinositide abundances relative to wild-type plants. Within the mutant, active auxin may be misdistributed but retain or exceed wild-type levels. In *pip5k1 pip5k2* double-mutant *A. thaliana* root tips, PtdIns(1,4)P2 levels are reduced. Although the abundance of auxin is unchanged, the distribution and polar transport of auxin are affected (Ischebeck *et al.*, 2013). Over-expression of PIP5K1 and PIP5K2 increase PtdIns(4,5)P2 levels. Roots have non-directional growth and have lost gravitropism, again suggesting perturbed auxin distribution (Ischebeck *et al.*, 2013). We suggest that active auxin levels are high within mutant internodes because during gravistimulation, auxin increases precede ethylene production within grass nodes (Wright *et al.*, 1978). The key ethylene biosynthetic enzyme, ACC oxidase (Lin *et al.*, 2009), is putatively encoded by GRMZM2G072529 and is up-regulated in the *bv1* mutants relative to wild-type plants. Possibly, apolar auxin distribution could contribute to the rectangular shapes of *bv1* internode parenchyma cells.

Genes misregulated within mutant internodes are notable for encoding cytoskeleton, cell wall, and membrane-associated proteins. GRMZM2G1187786 probably functions to maintain cytoskeleton function. It is expressed within wild-type internodes and silenced within the *bv1* mutants. GRMZM2G1187786 encodes a putative protein with 75% identity to the actin-related protein C3 (ARPC3; AT1G60430), which is one of seven subunits of the actin-related protein complex (Hussey *et al.*, 2006). In *A. thaliana*, the absence of functional ARP proteins causes shortened cells. Expanding cells contain dense actin bundles and exhibit pronounced morphological aberrations. (Li *et al.*, 2003; Mathur *et al*., 2003a,b; El-Assal *et al.*, 2004; Saedler *et al.*, 2004). Shortened *d50* mutant cells have actin thickened in longitudinally aligned bundles while wild-type rice internode parenchyma cells have a fine network of actin filaments (Zhong and Ye, 2004; Sato-Izawa *et al.*, 2012). Cell wall-related genes include GRMZM2G11834, GRMZM2G063917, and GRMZM2G103273. Polysaccharide-linked hydroxycinnamoyl esters ferulic acid and *p*-coumaric acid are important cell wall components and are produced by the phenylpropanoid pathway, of which PAL is the first committed step. Transcripts of two putative PAL-encoding genes, GRMZM2G118345 and GRMZM2G063917, are highly abundant in the *bv1* mutant relative to wild-type stems. The activity of PAL also greatly increases in *d50* (Nishikubo *et al.*, 2000; Sato-Izawa *et al.*, 2012), and the middle lamellae of *d50* cells have high levels of polysaccharide-linked ferulic acid and *p*-coumaric acids (Sato-Izawa *et al.*, 2012). GRMZM2G103273, a putative β-galactosidase, has low transcript abundance in mutant internodes relative to wild-type internodes. β-Galactosidases can target xyloglucan to regulate cellulose fibre separation during wall extension, and a blockage of xyloglucan digestion results in shortened plant organs within Arabidopsis (Sampedro *et al.*, 2012). GRMZM2G103273 is highly similar to the *A. thaliana* protein ATBGAL10 (BLASTP e-value 2e–25), which is responsible for the majority of β-galactose activity against xyloglucan. Misexpressed genes encoding membrane-associated transporters include GRMZM2G153920 and GRMZM2G180659. The sorbitol transporter-like protein GRMZM2G153920 is up-regulated in *bv1* relative to wild-type internodes. Sorbitol transporter-like proteins can transport pentoses as well as polyols and different hexoses (Klepek *et al.*, 2005). Cell wall-derived xylose and arabinose are high, and glucose is low in the Fukei 71/*d50* rice mutant relative to wild type (Nishikubo *et al.*, 2000). GRMZM2G180659 encodes a putative lysine histidine transporter, LHT1, and is similar to AUX transporters.

Although the substrates of BV1 are unknown, the putative functions of misexpressed genes suggest that BV1 contributes to plasma membrane-associated molecular transport. BV1 could affect transport by altering the patterns of vesicular cycling (Ischebeck *et al.*, 2013) and/or by altering the interaction of a phosphoinositide with an actin-binding protein, thereby affecting actin polymerization (Stevenson *et al.*, 2000). A high proportion of transcripts misexpressed within the *bv1* mutant internodes are co-expressed across maize development with genes whose functions are enriched for membrane-associated transport processes including glycolipid transport and amine transmembrane transport. Both *bv1* transcripts and many genes misregulated in *bv1* mutants accumulate in diverse maize tissues (Sekhon *et al.*, 2011; Downs *et al.*, 2013), indicating a broad function for BV1. We expect that paralogous 5PTases such as GRMZM2G065225 can compensate for the loss of BV1 function in tissues other than the expanding internode.

Variation among genes identified through the study of induced, large-effect mutations can contribute to quantitative trait variation within a population, and *bv1* may be allelic to plant architecture quantitative trait loci (QTLs) identified in mapping studies. In a segregating population derived from crosses between an Illinois High Oil population and an Illinois Low Oil population, Berke and Rocheford (1995) identified a QTL for plant height between markers npi449a and npi295a. This locus corresponds to a region between 151 and 169Mb on the maize physical map. Beavis *et al.* (1991) also identified a QTL for plant height in an F_2_ of inbred lines K05×W65 that maps close to *bv1*. Nonetheless, in an association mapping panel, Pfeiffer *et al.* (2014) found little correlation between *bv1* alleles and plant height. Similarly, SNP variation flanking the sorghum gene model homologous to *bv1*, Sobic.004G145500, was uncorrelated with plant height variation in a set of 377 diverse sorghum accessions (Casa *et al.*, 2008; X. Li and J. Yu, personal communication, 2015). We note that although BV1 affects the transcript abundance of GRMZM2G099467, a putative gibberellin 20 oxidase, this gene is not the orthologue of Os3g0122300, the gene responsible for the ‘green revolution’ dwarf rice. When using the rice green revolution protein in a BLASTP query, GRMZM2G180659 is the fifth best hit in the maize genome with 37.5% indentity over 315 aa, compared with 84% over 340 aa for the top hit.

Maize *bv1* alleles may have come under artificial selection, as has a sorghum allele of a *br2* homologue (Multani *et al.*, 2003), because stem lodging can significantly reduce maize yields (Elmore and Ferguson, 1999). Although our *bv1* alleles were very short and had smaller ears than wild-type plants, weak *bv1* alleles would be appealing targets for selection for a number of reasons. *bv1* mutants are indistinguishable from wild-type plants until after the transition of the apical meristem from the vegetative to the reproductive state and respond similarly to neighbouring plants and low R:FR light (Fig. 2 and Supplementary Figs S1 and S2). Although maize height and flowering time are consistently genetically correlated (Salas Fernandez *et al.*, 2009; Khanal *et al.*, 2011), *bv1* mutations do not change flowering times (Fig. 3C), a key trait for crop adaptation. *bv1* has normal flower development (data not shown) in contrast to other reduced-height maize mutants such as *anther ear1* (*an1*), *dwarf plant1* (*d1*), *dwarf plant3* (*d3*), *dwarf plant5* (*d5*), *dwarf plant8* (*d8*) and *dwarf plant11* (*d11*) (Fujioka et al., 1988a, b; Bensen *et al.*, 1995; Lawit *et al.*, 2010; Wang *et al.*, 2013). The *bv1* genomic region is not within the 256 regions identified by Heerwaarden *et al.* (2012) with evidence for directional selection as a product of modern, North America maize breeding. Nonetheless, we note that plant height in maize is a highly polygenic trait (Peiffer *et al.*, 2014), and selection could rapidly change height (or reduce lodging) with only moderate allele frequency shifts at many existing polymorphic loci, including *bv1*. In this case, association and selective sweep analyses would not identify these loci (Pritchard *et al.*, 2010). Suitable *bv1* alleles are candidates for future crop improvement.

## Supplementary data

Supplementary data are available at *JXB* online.

Fig. S1. Plant height of *bv1-1302* (bv1) and B73 (wt) plants grown with low R:FR reflected light (surrounding turfgrass) and high R:FR reflected light (no turfgrass) at different times after planting.

Fig. S2. Number of visible leaf tips of *bv1-1302* (bv1) and B73 (wt) plants grown with low R:FR reflected light (turfgrass) and high R:FR reflected light (no turfgrass).

Fig. S3. Internode lengths of field grown *bv1-1301*, *bv1-1302* and B73 plants measured 66 d after planting.

Fig. S4. Genotypes of plants with crossovers that mapped the *bv1* mutation to a ~97kb region on chromosome 5 (5:160 660 813–160 758 241).

Fig. S5. Multiple sequence alignment of maize GRMZM 2G366698_P01 subsequence and 20 homologous proteins across diverse plant species showing amino acid changes (in red rectangle) caused by the mutations in *bv1-1301* (A) and *bv1-1302* (B).

Table S1. Markers designed.

Table S2. Transcript abundance of GRMZM2G366698 across sample pools.

Table S3. Exon coding regions for the three potentially novel splice variants identified in the RNA-Seq reads.

Table S4. Number of differentially expressed genes between mutants *bv1-1301* and *bv1-1302* and wild-type genotypes *Bv1-1302* and *B73*.

Table S5. All 68 genes differentially expressed in *bv1-1301* and *bv1-1302* relative to *B73* (values in FPKM).

Supplementary Data
