# Supplementary material for: Brevis plant1, a putative inositol polyphosphate 5-phosphatase, is required for internode elongation in maize
Source: J Exp Bot. 2016 Jan 14;67(5):1577–88. doi: 10.1093/jxb/erv554 (PMC4762392; doi:10.1093/jxb/erv554)
Supplement: Supplementary Data [file supp_67_5_1577__index.html]

 Brevis plant1, a putative inositol polyphosphate 5-phosphatase, is required for internode elongation in maize — Supplementary Data 

# *Brevis plant1*, a putative inositol polyphosphate 5-phosphatase, is required for internode elongation in maize

## Supplementary Data

Data files

- supplementary\_tables\_S1\_S5\_figures\_S1\_S5 - Supplementary Data
