# Supplementary material for: Brevis plant1, a putative inositol polyphosphate 5-phosphatase, is required for internode elongation in maize
Source: J Exp Bot. 2016 Jan 14;67(5):1577–88. doi: 10.1093/jxb/erv554 (PMC4762392; doi:10.1093/jxb/erv554)
Supplement: Supplementary Data [file supp_erv554_supplementary_tables_S1_S5_figures_S1_S5.pdf]

Brevis plant1, a putative inositol polyphosphate 5-phosphatase, is required for internode elongation in maize (Zea mays L.)

Luis M Avila, Diego Cerrudo, Clarence Swanton, and Lewis Lukens

Supplemental File

Table S1. Markers designed
Table S2. Transcript abundance of GRMZM2G366698 across sample pools
Table S3. Exon coding regions for the three potentially novel splice variants identified in the RNA-Seq reads. The coding regions for annotated transcript Ref_GRMZM2G36669_T03 are listed for reference.
Table S4. Number of differentially expressed genes between mutants bv1-1301 and bv1-1302 and wild type genotypes Bv1-1302 and B73.
Table S5. All 68 genes differentially expressed in bv1-1301 and bv1-1302 relative to B73 (values in FPKM).

Figure S1. Plant height of bv1-1302 (bv1) and B73 (wt) plants grown with low R:FR reflected light (surrounding turfgrass) and high R:FR reflected light (no turfgrass) at different times after planting. Error bars depict the standard error.

Figure S2. Number of visible leaf tips of bv1-1302 (bv1) and B73 (wt) plants grown with low R:FR reflected light (turfgrass) and high R:FR reflected light (no turfgrass).

Figure S3. Internode lengths of field grown bv1-1301, bv1-1302 and B73 plants measured 66 days after planting. Error bars depict the standard error.

Figure S4. Genotypes of plants with crossovers that mapped the bv1 mutation to a ~97kb region on chromosome 5 (5:160,660,813-160,758,241). Phenotypes, genotypes and approximate physical locations of markers used are shown. Details for the markers close to and within GRMZM2G366698 are in Table S1.

Figure S5. Multiple sequence alignment of maize GRMZM2G366698_P01 subsequence and 20 homologous proteins across diverse plant species showing amino acid changes (in red rectangle) caused by the mutations in bv1-1301 (A) and bv1-1302 (B). Sequences are in descending order by percent of identity. E-value = 0 for all alignments. Amino acids are shaded according to similarity using the Blosum62 score matrix (black 100% similarity, dark grey 80%-100%, light grey 60-80%, white <60%)

Table S1. Markers designed

ID	Polymorphism type	Physicical position	Forward primer	Reverse primer	Anneaing temp °C	*Product length bp*
660A	SNP	5:160,660,813	CTCTACACCCACATCCCCAACT	ATCCTAATCATGTGCTTCTTCCA	61.2	468
758A	SNP	5:160,758,241	CACTCCCAGACTGTTGTTGTGTG	TAACTACAAGTGACCTGCCCTCT	61.2	382
721A	indel	5:160,721,861	GATGGAAGTGGAGAGGAAGAAGT	CCACTATCTAGCACGACCGAAG	64.4	200
Indel10	indel	5:160,710,364	GGCTTGTTTTGAGATCATTGG	AAGACTTGAAGATTCACATTCAGTAAA	60	230

Table S2. Transcript abundance of GRMZM2G366698 across sample pools.

Transcript	Nearest reference transcript	Length bp	B73 FPKM (c. i.)	*Bv1-1301* FPKM (c. i.)	*bv1-1301* FPKM (c. i.)	*bv1-1302* FPKM (c. i.)
GRMZM2G366698_T01	-	2648	4.06 (1.9; 6.2)	6.62 (3.9; 9.3)	1.59 (0.3; 2.9)	5.28 (2.9; 7.7)
potentially novel	GRMZM2G366698_T03	3646	1.46 (0.3; 2.6)	0.84 (0; 1.7)	1.15 (0.2; 2.1)	0.92 (0.0; 1.8)
potentially novel	GRMZM2G366698_T03	3396	2.01 (0.6; 3.4)	1.00 (0.1; 2.0)	0.00 (0; 0)	0.47 (0; 1.2)
potentially novel	GRMZM2G366698_T03	3651	0.93 (0; 1.9)	0.01 (0; 0)	2.89 (1.2; 4.5)	1.71 (0.4; 3.0)
GRMZM2G366698_T02	-	2664	1.00 (0; 2.2)	1.19 (0.1; 2.4)	3.34 (1.4; 5.3)	0.23 (0; 0.8)
GRMZM2G366698_T03	-	3236	0.00 (0; 0)	0.00 (0; 0)	0.00 (0; 0)	0.78 (0; 1.7)

*c. i. is the 95% confidence interval for FPKM values, as estimated by cufflinks' cuffdiff.

Table S3. Exon coding regions for the three potentially novel splice variants identified in the RNA-Seq reads. The coding regions for annotated transcript Ref_GRMZM2G36669_T03 are listed for reference.

chr	start	end	gene	transcript_id	exon	novel
5	160709566	160710041	GRMZM2G366698	GRMZM2G366698_T03_novel1	1	yes
5	160710373	160710703	GRMZM2G366698	GRMZM2G366698_T03_novel1	2	no
5	160710780	160710903	GRMZM2G366698	GRMZM2G366698_T03_novel1	3	no
5	160711057	160711306	GRMZM2G366698	GRMZM2G366698_T03_novel1	4	no
5	160711404	160711441	GRMZM2G366698	GRMZM2G366698_T03_novel1	5	no
5	160711730	160711895	GRMZM2G366698	GRMZM2G366698_T03_novel1	6	no
5	160716544	160716628	GRMZM2G366698	GRMZM2G366698_T03_novel1	7	no
5	160716707	160716828	GRMZM2G366698	GRMZM2G366698_T03_novel1	8	no
5	160717309	160718113	GRMZM2G366698	GRMZM2G366698_T03_novel1	9	no
5	160718209	160718458	GRMZM2G366698	GRMZM2G366698_T03_novel1	10	yes
5	160721062	160722065	GRMZM2G366698	GRMZM2G366698_T03_novel1	11	yes
5	160709566	160710041	GRMZM2G366698	GRMZM2G366698_T03_novel2	1	yes
5	160710373	160710703	GRMZM2G366698	GRMZM2G366698_T03_novel2	2	no
5	160710780	160710903	GRMZM2G366698	GRMZM2G366698_T03_novel2	3	no
5	160711057	160711306	GRMZM2G366698	GRMZM2G366698_T03_novel2	4	no
5	160711404	160711441	GRMZM2G366698	GRMZM2G366698_T03_novel2	5	no
5	160711730	160711895	GRMZM2G366698	GRMZM2G366698_T03_novel2	6	no
5	160716544	160716628	GRMZM2G366698	GRMZM2G366698_T03_novel2	7	no
5	160716712	160716828	GRMZM2G366698	GRMZM2G366698_T03_novel2	8	yes
5	160717309	160718113	GRMZM2G366698	GRMZM2G366698_T03_novel2	9	no
5	160721062	160722065	GRMZM2G366698	GRMZM2G366698_T03_novel2	10	yes
5	160709566	160710041	GRMZM2G366698	GRMZM2G366698_T03_novel3	1	yes
5	160710373	160710703	GRMZM2G366698	GRMZM2G366698_T03_novel3	2	no
5	160710780	160710903	GRMZM2G366698	GRMZM2G366698_T03_novel3	3	no
5	160711057	160711306	GRMZM2G366698	GRMZM2G366698_T03_novel3	4	no
5	160711404	160711441	GRMZM2G366698	GRMZM2G366698_T03_novel3	5	no
5	160711730	160711895	GRMZM2G366698	GRMZM2G366698_T03_novel3	6	no
5	160716544	160716628	GRMZM2G366698	GRMZM2G366698_T03_novel3	7	no
5	160716712	160716828	GRMZM2G366698	GRMZM2G366698_T03_novel3	8	yes
5	160717309	160718113	GRMZM2G366698	GRMZM2G366698_T03_novel3	9	no
5	160718209	160718458	GRMZM2G366698	GRMZM2G366698_T03_novel3	10	yes
5	160721062	160722065	GRMZM2G366698	GRMZM2G366698_T03_novel3	11	yes
5	160709628	160710041	GRMZM2G366698	Ref_GRMZM2G36669_T03	1	-
5	160710373	160710703	GRMZM2G366698	Ref_GRMZM2G36669_T03	2	-
5	160710780	160710903	GRMZM2G366698	Ref_GRMZM2G36669_T03	3	-
5	160711057	160711306	GRMZM2G366698	Ref_GRMZM2G36669_T03	4	-
5	160711404	160711441	GRMZM2G366698	Ref_GRMZM2G36669_T03	5	-
5	160711730	160711895	GRMZM2G366698	Ref_GRMZM2G36669_T03	6	-
5	160716544	160716628	GRMZM2G366698	Ref_GRMZM2G36669_T03	7	-
5	160716707	160716828	GRMZM2G366698	Ref_GRMZM2G36669_T03	8	-
5	160717309	160718113	GRMZM2G366698	Ref_GRMZM2G36669_T03	9	-
5	160721062	160721962	GRMZM2G366698	Ref_GRMZM2G36669_T03	10	-

Table S4. Number of differentially expressed genes between mutants *bv1-1301* and *bv1-1302* and wild type genotypes *Bv1-1302* and B73.

	bv1-1301	***bv1-1302***	***Bv1-1302***	**B73**
bv1-1301	0			
bv1-1302	98	0		
Bv1-1302	469	239	0	
**B73**	256	108	58	0

Table S5. All 68 genes differentially expressed in *bv1-1301* and *bv1-1302* relative to B73 (values in FPKM).

Gene id	B73	Bv1-1302	*bv1-1301*	*bv1-1302*	c. module ; module membership
GRMZM2G066805	1.17	2.089	0.00	0.00	
GRMZM2G099467	3.27	1.81	81.31	43.81	Zm_mod07; 0.70
GRMZM2G090043*	0.00	0.35	37.85	37.84	Zm_mod14; -0.76
GRMZM5G809218	11.22	8.25	67.39	41.59	
GRMZM2G153920	0.00	0.00	1.15	0.98	Zm_mod04; -0.49
GRMZM2G050514*	22.93	8.74	4.13	6.61	
GRMZM2G072529	0.00	0.00	2.29	1.78	
GRMZM2G050234*	2.00	0.53	33.63	14.23	Zm_mod02; -0.66
AC218093.3_FG005	10.43	20.56	0.00	0.00	
GRMZM2G118345	2.62	3.34	21.66	16.10	Zm_mod07; 0.71
GRMZM5G847274*	25.62	3.67	2.71	2.21	
GRMZM2G048033	1.44	1.62	0.00	0.00	
GRMZM2G088819*	0.00	0.11	6.95	3.29	Zm_mod07; 0.79
GRMZM5G868679*	2.98	3.96	12.60	11.43	
GRMZM2G021020	22.24	22.90	0.00	0.00	
GRMZM2G141693	3.68	9.26	0.00	0.00	
GRMZM2G072493	7.11021	18.1459	0.00	0.00	Zm_mod03; 0.29
GRMZM5G885061	1.36534	3.93228	0.00	0.00	
GRMZM2G089574*	3.83733	0.00	0.00	0.00	
GRMZM2G135013	1.87	2.12	12.22	11.66	Zm_mod11; 0.92
GRMZM2G015921	4.40	7.22	0.00	0.00	
GRMZM2G177991	0.00	0.00	2.40	4.72	Zm_mod16; 0.99
GRMZM2G066870*	0.00	0.10	22.82	5.59	Zm_mod07; 0.64
GRMZM2G127789	0.00	0.00	4.15755	2.81	
GRMZM5G871347*	0.00	0.17	1.26	1.01	
GRMZM5G869453	9.17	24.01	0.00	0.00	
GRMZM2G314954	2.14	3.41	0.00	0.00	

GRMZM2G008283	15.17	33.16	0.00	0.00	
AC209784.3_FG007	4.55	4.57	18.79	17.44	
GRMZM2G092474*	0.00	0.60	9.86	6.49	
GRMZM2G159908*	1.12	1.44	18.17	13.44	Zm_mod07; 0.9
GRMZM2G103273	3.39	5.94	0.00	0.00	
GRMZM2G003970	4.31	5.28	77.30	40.49	Zm_mod13; -0.94
GRMZM2G063917	0.00	0.00	8.52	2.83	Zm_mod05; 0.62
GRMZM2G020508	4.44	4.11	12.96	13.87	
GRMZM2G017557*	1.08	0.09	0.00	0.00	Zm_mod11; 0.84
GRMZM2G134708	4.89	3.57	34.15	24.52	Zm_mod07; 0.62
GRMZM2G180775*	0.00	0.20	1.29	1.64	Zm_mod27; -0.50
GRMZM2G007729*	20.11	31.54	102.32	59.32	Zm_mod04; -0.47
GRMZM2G130389	2.09	1.85	26.58	18.79	
GRMZM2G104783*	90.47	79.41	41.05	43.27	Zm_mod09; -0.54
GRMZM2G049021	12.61	0.00	42.65	39.69	
GRMZM2G129189	0.00	0.00	2.82	1.93	Zm_mod07; 0.59
GRMZM2G170588	4.60	8.35	0.00	0.00	
GRMZM2G347174	0.00	0.00	9.01	6.77	
GRMZM2G118786	0.89	1.31	0.00	0.00	
GRMZM2G176206	1.11	0.64	10.54	8.72	Zm_mod04; -0.80
GRMZM2G334660*	0.00	0.22	3.33	2.21	
GRMZM2G151204*	0.00	0.30	1.35	1.00	Zm_mod00; 0
GRMZM2G007195*	9.62	11.47	39.84	28.26	
AC209636.2_FG003*	0.00	0.00	2.28	1.69	
GRMZM5G892685*	7.17	5.57	65.45	36.99	
GRMZM2G180659	2.27	2.83	36.45	27.36	Zm_mod07; 0.82
GRMZM2G126302	2.57	3.20	0.00	0.00	
GRMZM2G410815	19.04	0.00	76.01	68.29	
GRMZM2G117281	36.66	28.65	369.56	303.77	
GRMZM2G306371	2.53	5.26	0.00	0.00	

GRMZM2G039757*	1.88	2.56	19.23	13.49	Zm_mod03; -0.52
GRMZM2G044194*	0.00	0.70	9.75	7.50	Zm_mod09; 0.63
GRMZM2G172204	59.79	58.61	503.74	489.16	Zm_mod03; 0.55
GRMZM2G322836*	1.14	0.71	0.00	0.00	
GRMZM2G169149	0.00	0.00	10.87	3.91	
GRMZM2G089493*	1.61	0.21	0.00	0.00	
GRMZM2G331393	0.00	0.00	1.69	1.92	
GRMZM5G802801	12.65	13.53	49.64	32.22	
GRMZM2G094510	14.43	8.21	79.62	58.74	Zm_mod08; -0.28
GRMZM2G037485*	0	0.42	3.59	4.97	Zm_mod07; 0.61
Novel assembly, no gene ID	1.22	0.97	0	0	

* Not differentially expressed between *bv1_1302* and *Bv1_1302*.

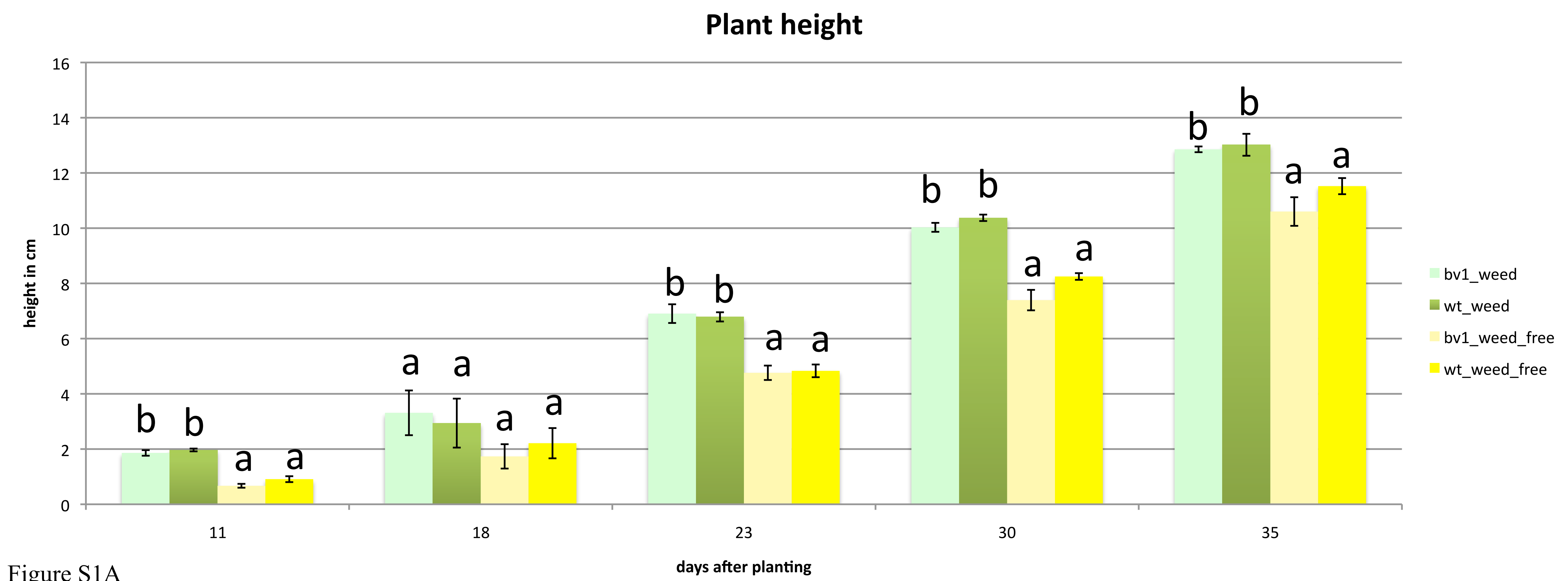
Plant height
height in cm
16
14
12
10
8
6
4
2
0
11
18
23
30
35
days after planting
b b a a
a a a a
b b a a
b b a a
b b a a
bv1_weed
wt_weed
bv1_weed_free
wt_weed_free

Figure S1A

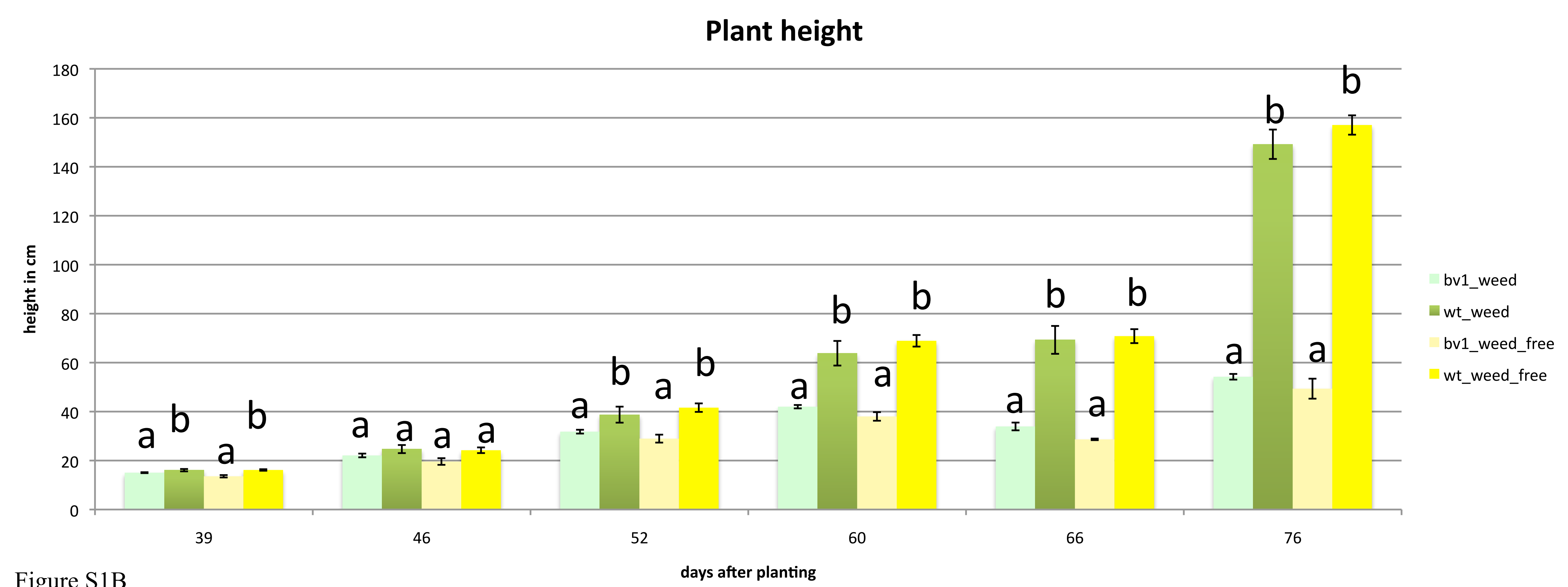

Plant height
height in cm
180
160
140
120
100
80
60
40
20
0
39
46
52
60
66
76
days after planting
bv1_weed
wt_weed
bv1_weed_free
wt_weed_free

Figure S1B

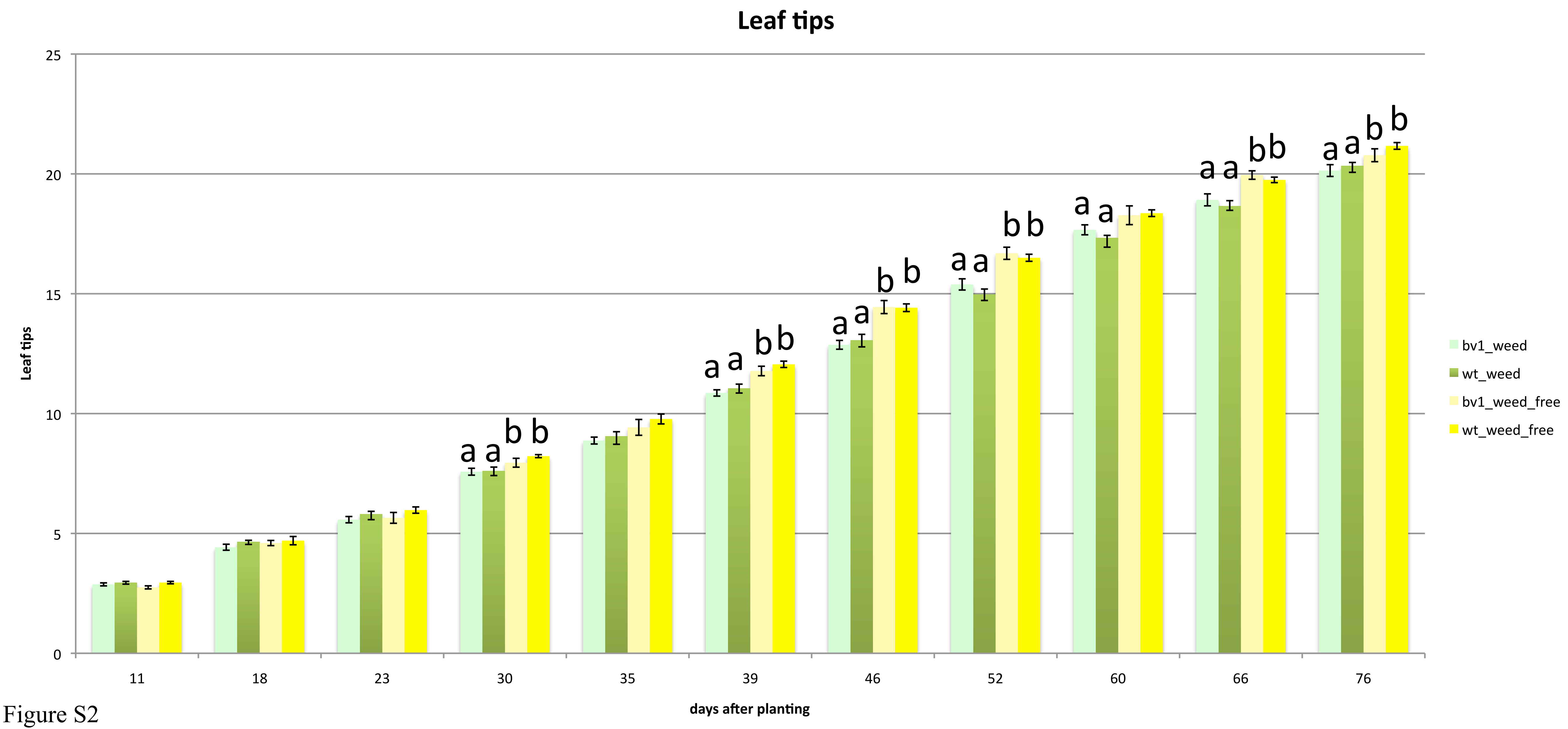
Leaf tips
25
20
15
10
5
0
Leaf tips
11
18
23
30
35
39
46
52
60
66
76
days after planting
a a b b
a a b b
a a b b
a a b b
a a
a a bb
a a b b
bv1_weed
wt_weed
bv1_weed_free
wt_weed_free

Figure S2

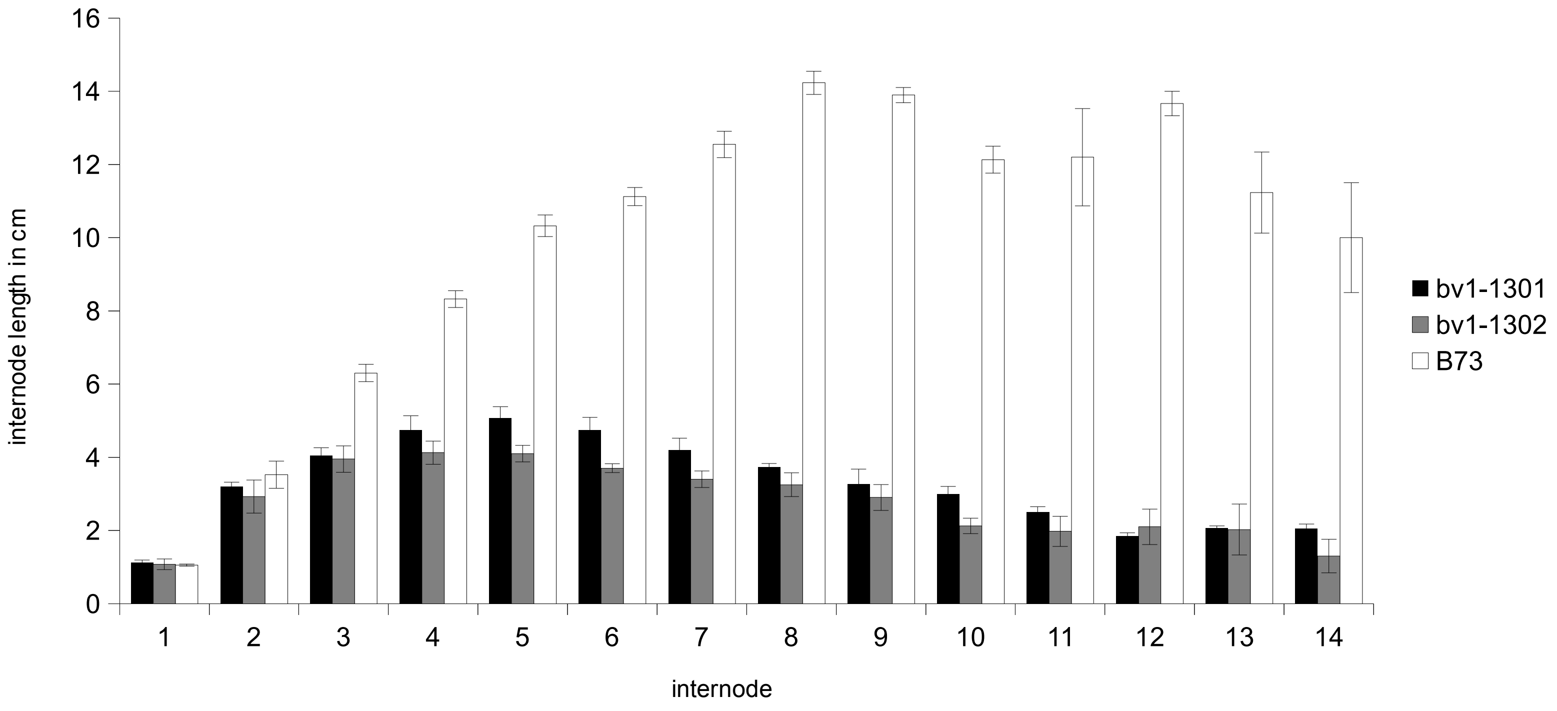
16
14
12
10
8
6
4
2
0
internode length in cm
1
2
3
4
5
6
7
8
9
10
11
12
13
14
internode
bv1-1301
bv1-1302
B73

Figure S3

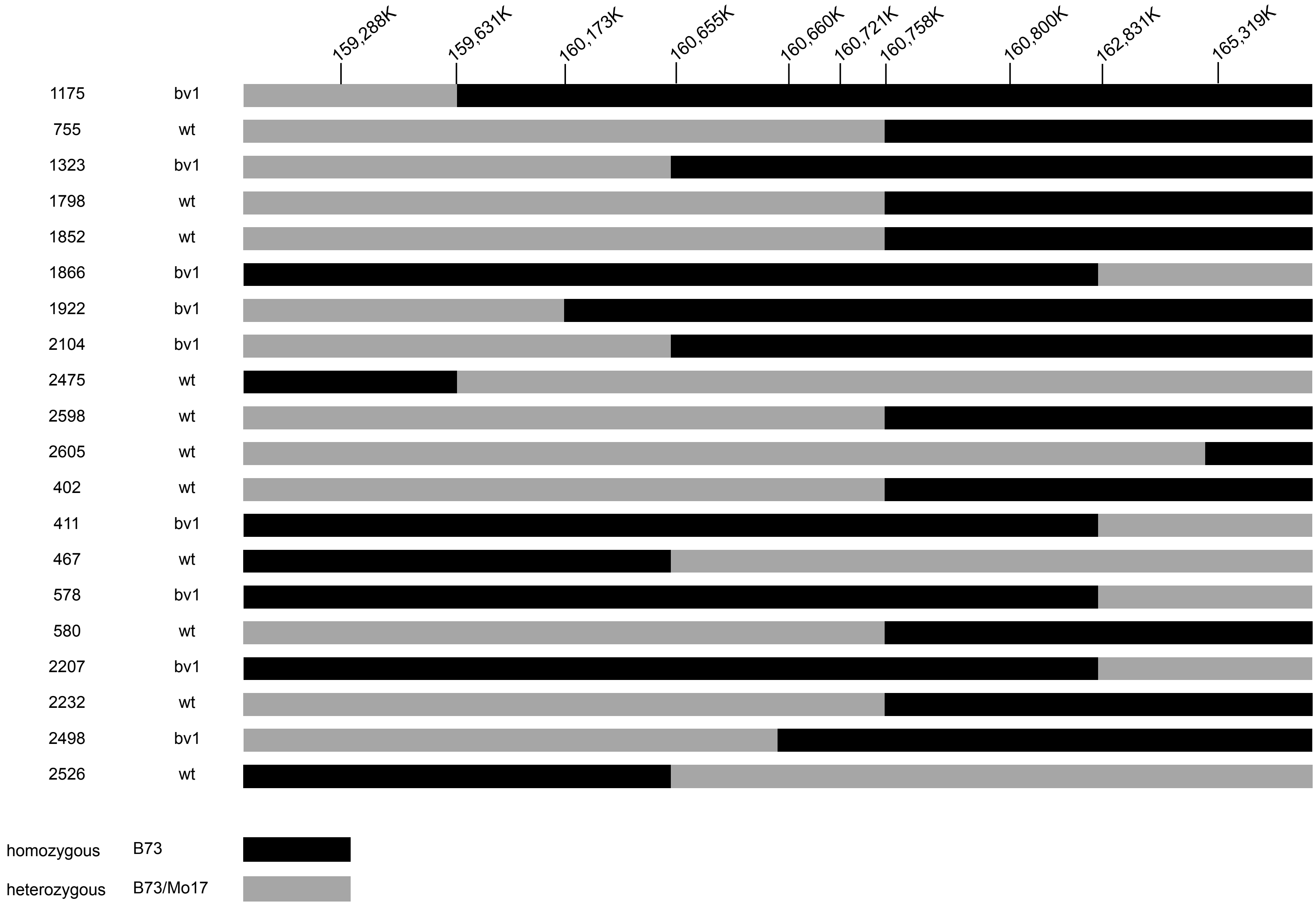
159,288K
159,631K
160,173K
160,655K
160,660K
160,721K
160,758K
160,800K
162,831K
165,319K
1175 bv1
755 wt
1323 bv1
1798 wt
1852 wt
1866 bv1
1922 bv1
2104 bv1
2475 wt
2598 wt
2605 wt
402 wt
411 bv1
467 wt
578 bv1
580 wt
2207 bv1
2232 wt
2498 bv1
2526 wt
homozygous B73
heterozygous B73/Mo17

Figure S4

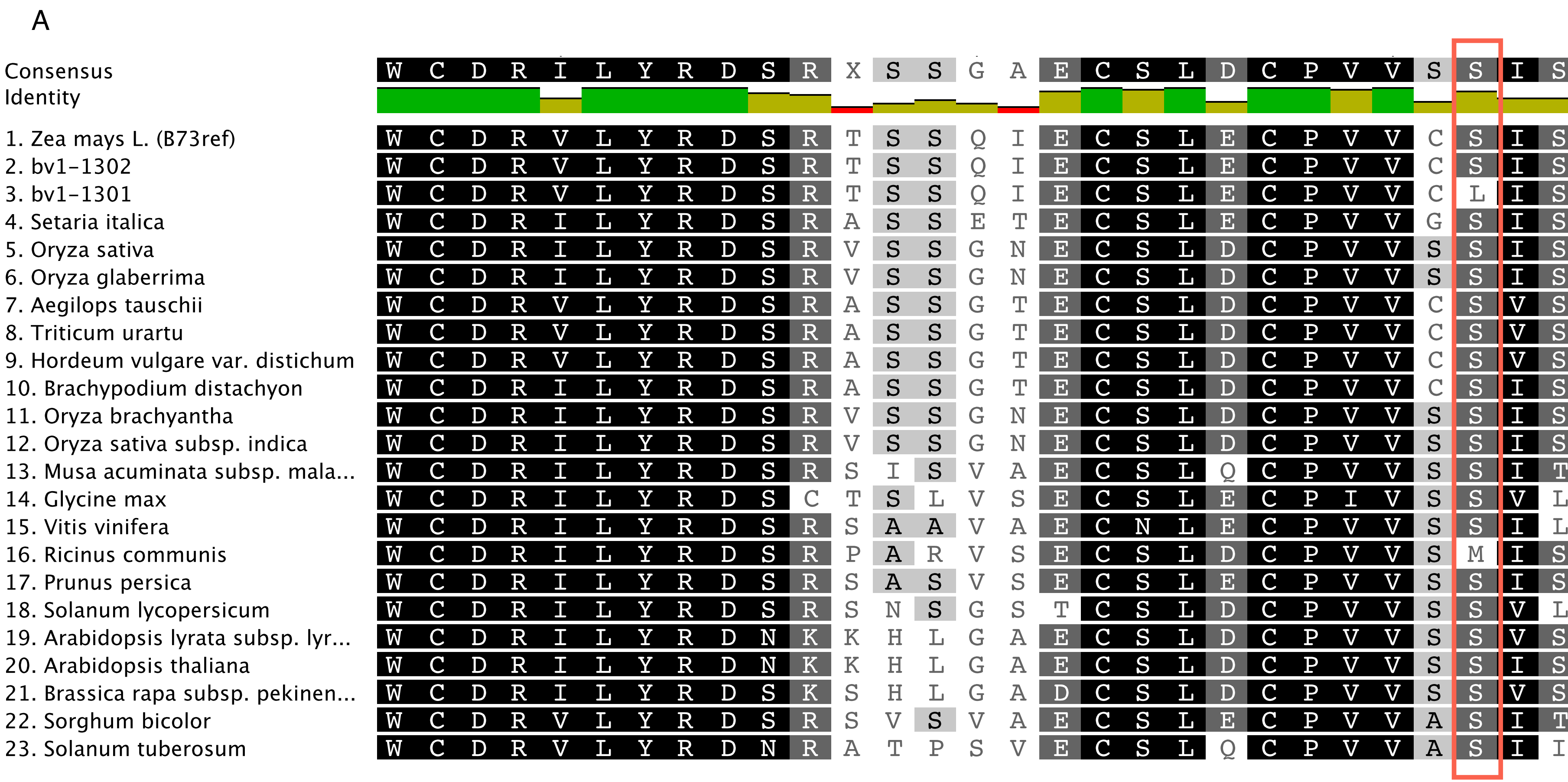
A
Consensus W C D R I L Y R D S R X S S G A E C S L D C P V V S S I S
Identity
1. Zea mays L. (B73ref) W C D R V L Y R D S R T S S Q I E C S L E C P V V C S I S
2. bv1-1302 W C D R V L Y R D S R T S S Q I E C S L E C P V V C S I S
3. bv1-1301 W C D R V L Y R D S R T S S Q I E C S L E C P V V C L I S
4. Setaria italica W C D R I L Y R D S R A S S E T E C S L E C P V V G S I S
5. Oryza sativa W C D R I L Y R D S R V S S G N E C S L D C P V V S S I S
6. Oryza glaberrima W C D R I L Y R D S R V S S G N E C S L D C P V V S S I S
7. Aegilops tauschii W C D R V L Y R D S R A S S G T E C S L D C P V V C S V S
8. Triticum urartu W C D R V L Y R D S R A S S G T E C S L D C P V V C S V S
9. Hordeum vulgare var. distichum W C D R V L Y R D S R A S S G T E C S L D C P V V C S V S
10. Brachypodium distachyon W C D R I L Y R D S R A S S G T E C S L D C P V V C S I S
11. Oryza brachyantha W C D R I L Y R D S R V S S G N E C S L D C P V V S S I S
12. Oryza sativa subsp. indica W C D R I L Y R D S R V S S G N E C S L D C P V V S S I S
13. Musa acuminata subsp. mala... W C D R I L Y R D S R S I S V A E C S L Q C P V V S S I T
14. Glycine max W C D R I L Y R D S C T S L V S E C S L E C P I V S S V L
15. Vitis vinifera W C D R I L Y R D S R S A A V A E C N L E C P V V S S I L
16. Ricinus communis W C D R I L Y R D S R P A R V S E C S L D C P V V S M I S
17. Prunus persica W C D R I L Y R D S R S A S V S E C S L E C P V V S S I S
18. Solanum lycopersicum W C D R I L Y R D S R S N S G S T C S L D C P V V S S V L
19. Arabidopsis lyrata subsp. lyr... W C D R I L Y R D N K K H L G A E C S L D C P V V S S V S
20. Arabidopsis thaliana W C D R I L Y R D N K K H L G A E C S L D C P V V S S I S
21. Brassica rapa subsp. pekinen... W C D R I L Y R D S K S H L G A D C S L D C P V V S S V S
22. Sorghum bicolor W C D R V L Y R D S R S V S V A E C S L E C P V V A S I T
23. Solanum tuberosum W C D R V L Y R D N R A T P S V E C S L Q C P V V A S I I

Figure S5A

B

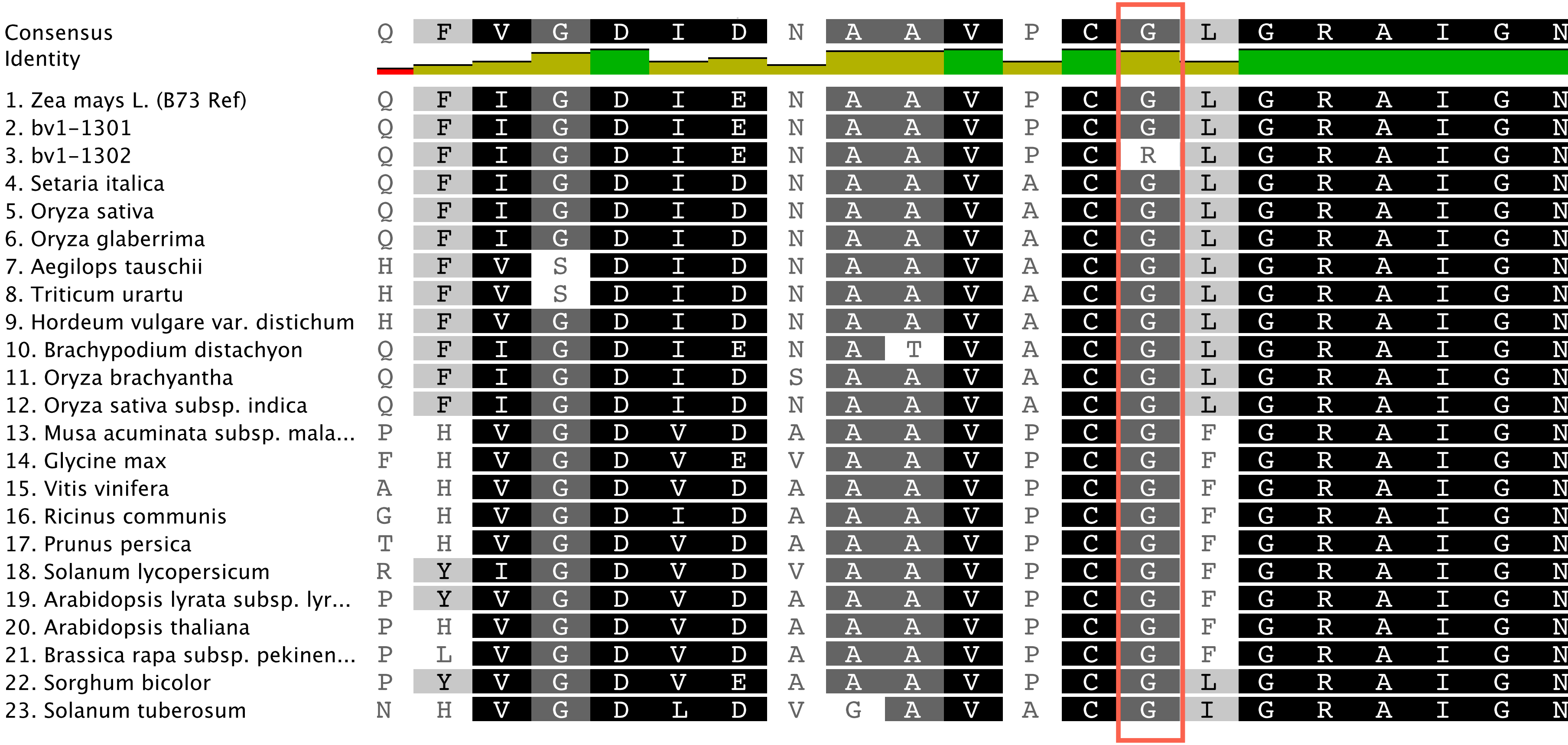
Consensus Q F V G D I D N A A V P C G L G R A I G N
Identity
1. Zea mays L. (B73 Ref) Q F I G D I E N A A V P C G L G R A I G N
2. bv1-1301 Q F I G D I E N A A V P C G L G R A I G N
3. bv1-1302 Q F I G D I E N A A V P C R L G R A I G N
4. Setaria italica Q F I G D I D N A A V A C G L G R A I G N
5. Oryza sativa Q F I G D I D N A A V A C G L G R A I G N
6. Oryza glaberrima Q F I G D I D N A A V A C G L G R A I G N
7. Aegilops tauschii H F V S D I D N A A V A C G L G R A I G N
8. Triticum urartu H F V S D I D N A A V A C G L G R A I G N
9. Hordeum vulgare var. distichum H F V G D I D N A A V A C G L G R A I G N
10. Brachypodium distachyon Q F I G D I E N A T V A C G L G R A I G N
11. Oryza brachyantha Q F I G D I D S A A V A C G L G R A I G N
12. Oryza sativa subsp. indica Q F I G D I D N A A V A C G L G R A I G N
13. Musa acuminata subsp. mala... P H V G D V D A A A V P C G F G R A I G N
14. Glycine max F H V G D V E V A A V P C G F G R A I G N
15. Vitis vinifera A H V G D V D A A A V P C G F G R A I G N
16. Ricinus communis G H V G D I D A A A V P C G F G R A I G N
17. Prunus persica T H V G D V D A A A V P C G F G R A I G N
18. Solanum lycopersicum R Y I G D V D V A A V P C G F G R A I G N
19. Arabidopsis lyrata subsp. lyr... P Y V G D V D A A A V P C G F G R A I G N
20. Arabidopsis thaliana P H V G D V D A A A V P C G F G R A I G N
21. Brassica rapa subsp. pekinen... P L V G D V D A A A V P C G F G R A I G N
22. Sorghum bicolor P Y V G D V E A A A V P C G L G R A I G N
23. Solanum tuberosum N H V G D L D V G A V A C G I G R A I G N

Figure S5B
